# Xenodiagnosis of *Leishmania donovani* in BALB/c mice using *Phlebotomus orientalis*: a new laboratory model

**DOI:** 10.1186/s13071-015-0765-x

**Published:** 2015-03-14

**Authors:** Jovana Sadlova, Veronika Seblova, Jan Votypka, Alon Warburg, Petr Volf

**Affiliations:** Department of Parasitology, Faculty of Science, Charles University in Prague, Prague 2, Vinicna 7, 128 44 Czech Republic; Department of Microbiology & Molecular Genetics, The Institute for Medical Research Israel-Canada, The Kuvin Centre for the Study of Infectious & Tropical Diseases, The Hebrew University - Hadassah Medical School, The Hebrew University of Jerusalem, Jerusalem, 91120 Israel

**Keywords:** Xenodiagnosis, Visceral leishmaniasis, Phlebotomine sand flies, Asymptomatic reservoir hosts, Vector-borne diseases, *Leishmania donovani*

## Abstract

**Background:**

In areas endemic for visceral leishmaniasis (VL), the majority of infected hosts remain asymptomatic but potentially infectious to biting sand flies. Their infectiousness for sand fly vectors is crucial for the transmission of the disease and can be quantified only by xenodiagnosis. However, in the case of human hosts, xenodiagnosis can be problematic for ethical and logistic reasons. The BALB/c mouse model described in this paper was designed to enable xenodiagnostic studies on VL hosts circumventing the need for human volunteers, it permits xenodiagnosis using the same individual host repeatedly, over several months.

**Methods:**

BALB/c mice were intradermally inoculated in the ear pinnae with *Leishmania donovani*, primarily metacyclic stages isolated from the thoracic midguts of experimentally-infected *Phlebotomus orientalis* females. Naïve sand flies were allowed to feed on anaesthetized mice in 1-3-weeks- interval, firstly on the site of inoculation of *L. donovani* (weeks 2–8 post infection, p.i.), later on the whole body of mice (weeks 9 – 15 p.i.). Infections of sand flies were evaluated microscopically or by PCR analysis.

**Results:**

Although infected mice did not show any signs of disease, 19% (N = 876) of the *P. orientalis* females that fed at the site of inoculation, became infected. The majority of *L. donovani*-positive females (76%) had heavy infections with their stomodeal valves colonized by attached parasites. Inoculated mouse ears remained infective for sand flies until week 15 p.i. Females feeding on other parts of the body remained negative with exception of two groups feeding on contralateral ears by week 12 p.i. On week 15, however, these two mice returned negative at xenodiagnosis of the contralateral ears. In sacrificed mice, the highest parasite numbers were found in inoculated ears and their draining lymph nodes. Infections were detected also in the spleen, liver, blood and rarely in the contralateral ear.

**Conclusions:**

The study showed that BALB/c mice harbored parasites in sufficient numbers to promote heavy infections in *P. orientalis* and thus comprised a suitable laboratory model for xenodiagnoses of *L. donovani*. Parasites persisted in the inoculation site and were found transmissible for months to sand flies biting on the same site.

## Background

Visceral leishmaniasis (VL), also known as kala-azar, is caused by *Leishmania* parasites belonging to the *L. donovani* complex (Kinetoplastida: Trypanosomatidae) and transmitted by phlebotomine sand flies (Diptera: Psychodidae). The disease is prevalent among mainly poor rural and suburban populations in Asia, Africa and Latin America. An estimated 390,000 VL cases occur annually, over 90% of which are concentrated in the Indian sub-continent, East Africa and Brazil [1]. Epidemiology of two causative agents of the disease, *L. donovani* and *L. infantum*, significantly differs; while *L. infantum* circulates as zoonosis with dogs serving as the main reservoir hosts, closely related *L. donovani* is mostly assumed to be anthroponotic [1,2].

VL caused by *L. donovani* is a serious systemic disease but most humans infected with *L. donovani* remain asymptomatic – i.e. positive by serology, polymerase chain reaction (PCR) and/or Leishmanin Skin Test (LST), but free of disease. The ratio of asymptomatic to patent infections ranges from 1:2.4 to 5.6:1 in East Africa and from 4:1 to 9:1 in the Indian subcontinent (reviewed by [3]) Importantly, asymptomatic individuals with amastigotes in their blood or skin are potentially infectious to biting sand flies and may serve as “cryptic” reservoir hosts.

A crucial factor in the spread of vector-borne diseases is the propensity of infected hosts to serve as parasite reservoirs for the vectors. Moreover, for the implementation of effective control of VL it is essential to estimate to what extent asymptomatic hosts are responsible for infecting the vector sand flies. Mathematical modeling based on data from the Indian subcontinent indicated that transmission of *L. donovani* is predominantly driven by asymptomatic persons, whose incidence is much higher than symptomatic VL cases [4]. A cohort study conducted in north Ethiopia calculated that only about 3.2% of the asymptomatic human carriers of *L. donovani* (k-DNA qPCR positive) had sufficiently high parasitaemias (>1000 parasites per ml of blood) to infect sand flies efficiently. These asymptomatic carriers with high parasitaemias were mathematically estimated to be responsible for about 65% of the infected sand flies [5]. It should be noted that in highly susceptible vectors like *P. orientalis*, as few as one or two parasites per bloodmeal were demonstrated to establish infection in 50% of sand fly females [6].

The optimal method for testing the infectiousness of hosts to biting vectors is by xenodiagnosis. Such studies have been performed predominantly with *L. infantum*. Many studies on dogs described the correlation between clinical symptoms and infectiousness to biting sand flies. Meta-analysis of these studies showed that asymptomatic dogs infect similar proportions of biting sand flies as do symptomatic ones [7]. In humans, two xenodiagnostic studies have been performed. The study on human volunteers showed that Brazilian VL cases were infectious to feeding sand fly females while volunteers who were asymptomatic, were not [8]. However, in this study, asymptomatic infections were identified using LST that detects only late-stage cell-mediated immunity [9]. Hence, these LST positive volunteers may have already harbored low parasitaemias or could have even been parasite-free. The second study by Molina et al. [10] found both asymptomatic and symptomatic patients infective for *Phlebotomus perniciosus* sand flies.

Several studies on cutaneous leishmaniasis (CL) have established that sand fly bites result in infections characterized by typical immune reactions and distinct pathologies from infections delivered by injection [11-13]. Clearly transmission of the parasite is the crucial stage for infecting the hosts; however, the transmission dynamics of the disease are affected much more significantly by the infectiousness of the hosts for naïve sand flies. While an individual host can only be infected once, it may infect a large number of sand flies for prolonged periods. Hence the infectiousness of the host is the crucial driver of transmission in vector-borne diseases [5].

*Leishmania donovani s. str*. is assumed to circulate anthroponotically among humans; a role for animal reservoir hosts has been suggested but not conclusively proven. Performing xenodiagnostic studies with human volunteers is highly problematic for ethical and logistical reasons. Therefore, we established a BALB/c mouse model for xenodiagnosis of VL circumventing the need for human volunteers. Using the mouse model we were able to (i) perform xenodiagnosis with the same host repeatedly during several months and (ii) expose the whole body of the host to biting sand flies. This facilitated the collection of data about temporal and spatial patterns of infectiousness of mice without external signs of disease for sand flies. Such data may promote improved understanding of the transmission dynamics and epidemiology of VL.

This is the first paper to characterize the infectiousness profile for biting sand flies of hosts without external signs of disease infected with *L. donovani*. BALB/c mice were intradermally inoculated with *L. donovani* and exposed to biting *P. orientalis*, the vector of VL in north Ethiopia and Sudan [14]. Like asymptomatic human cases, experimentally infected mice harbored *L. donovani* parasites for many weeks without showing any apparent symptoms. We studied the time course of the infectiousness of mice for sand flies and compared the relative proportions of sand flies infected by feeding on different parts of the body.

## Methods

### Sand flies and parasites

*Phlebotomus orientalis* colony was maintained under standard conditions (26°C on 50% sucrose and 14 h light/10 h dark photoperiod) as described previously [15]. *Leishmania donovani* (MHOM/ET/2010/GR374) was cultured in M199 medium (Sigma) containing 10% heat-inactivated fetal calf serum (Gibson) supplemented as described by Sadlova et al. [16]. Cultivations of parasites from mouse spleens were done on blood agar slopes overlaid with the same medium.

### Infections of BALB/c mice with sand fly-derived *Leishmania*

*P. orientalis* females were infected with *L. donovani* by feeding through chick-skin membranes on heparinized rabbit blood containing 10^6^ promastigotes/ml. Engorged females were maintained at 26°C on 50% sucrose and dissected on day 10 or 12 post bloodmeal (PBM); their midguts were checked microscopically for the presence of promastigotes and thoracic midguts (the site of accumulation of metacyclic forms) with good density of parasites, were pooled in sterile saline. Pools of freshly dissected 70 thoracic midguts were homogenized in 35 μl saline. Immediately, BALB/c mice anaesthetized with ketamin/xylazin (150 mg and 15 mg/kg, respectively) were injected with 5 μl of the parasite suspension intradermally into the inner sides of ear pinnae using syringe. Exact numbers of all parasites stages were calculated using a Burker apparatus and the proportions of metacyclic forms was identified on Giemsa stained smears based on morphological criteria described previously [17]. In two repeats of the experiment, *L. donovani* numbers in the inoculums were 1.4 × 10^5^ and 9.3 × 10^4^ per mouse, metacyclics comprised 65% and 73% of all forms, i.e., 9.1 × 10^4^ and 6.8 × 10^4^ per mouse. Mice were checked weekly for external signs of the disease till week 8 or 15 post infection (p.i.) when they were sacrificed.

### Xenodiagnosis I

The first series of xenodiagnosis experiments was performed between weeks 2 and 8 p.i. Five to seven-day-old *P. orientalis* females were allowed to feed on the inoculated ear (the site of inoculation of *L. donovani*) of anaesthetized mice in two-weeks- interval. Ten mice were exposed to sand flies in two repeats of the experiment (four and six mice in particular experiments). Two mice were exposed in the same cage to approximately 200 sand fly females and values from each pair were summed. Fed sand fly females were separated and maintained at 26°C on 50% aqueous sucrose solution. Eight days PBM females were dissected and their guts examined under the light microscope. Intensities and locations of infections were evaluated as described previously [18].

### Xenodiagnosis II

The second series of xenodiagnosis using five mice infected with *L. donovani* was done between weeks 9 and 15 p.i. *P. orientalis* females were allowed to feed on the whole body of anesthetized mice, fed females were picked out based on where they had fed and separated according to the place of feeding to four groups (a) inoculated ear, (b) contralateral ear, (c) front paws, (d) hind paws together with tail. Females were maintained on 50% aqueous sucrose solution at 26°C till day 2 PBM and then stored at −20°C for the subsequent analysis by qPCR. During experimental xenodiagnosis II, fed sand fly females were tested for the presence of *Leishmania* parasites on day 2 PBM. This shorter time interval decreased the mortality of sand fly females (from about 55% by day 8 to 8% on day 2 PBM). Sand flies fed on the inoculated ear were tested individually; sand flies fed on other parts of mice body were pooled for PCR analysis. The qPCR detection was chosen as it is an accurate method to determine the true numbers of parasites within the bloodmeal [18].

### Mice sampling and quantitative PCR

Five mice (No 3, 4, 6, 8 and 10) were sacrificed eight weeks p.i., remaining five mice (No. 1, 2, 5, 7, 9) were sacrificed 15 weeks p.i. by injecting them an overdose of ketamin/xylazin anaesthesia. Both ears (inoculated and contralateral), both ear-draining lymph nodes, spleen, liver, paws, tail and in 6 mice also blood were stored at −20°C for qPCR. Extraction of total DNA from mice tissues and sand flies was performed using a DNA tissue isolation kit (Roche Diagnostics, Indianapolis, IN) according to the manufacturer’s instructions. Q PCR for detection and quantification of *Leishmania* parasites was performed in Bio-Rad iCycler&iQ Real-Time PCR Systems using the SYBR Green detection method (iQ SYBR Green Supermix, Bio-Rad, Hercules, CA). For adequate sensitivity, minicircle kinetoplast DNA (kDNA) was chosen as the molecular target, using the primers described by [16] (forward primer 5′-CTTTTCTGGTCCTCCGGGTAGG-3′ and reverse primer 5′-CCACCCGGCCCTATTTTACACCAA-3′).

### Ethical considerations

Animals were maintained and handled in the animal facility of Charles University in Prague in accordance with institutional guidelines and Czech legislation (Act No. 246/1992 and 359/2012 coll. on Protection of Animals against Cruelty in present statutes at large), which complies with all relevant European Union and international guidelines for experimental animals. All the experiments were approved by the Committee on the Ethics of Laboratory Experiments of the Charles University in Prague and were performed under permission no. MSMT-31114/2013-13 of the Ministry of the Environment of the Czech Republic. Investigators are certificated for experimentation with animals by the Ministry of Agriculture of the Czech Republic.

## Results

### Xenodiagnosis experiment I: week 2 – 8 post inoculation

All of the ten mice infected in their ear pinnae with *L. donovani* did not show any external signs of the disease throughout the entire experiment; no weight loss, dehydration, alopecia, hunched posture and/or scabs or sores were observed in any experimental animal. *P. orientalis* females were allowed to feed at the site of parasite inoculation on weeks 2, 4, 6 and 8 post infection (p.i). In total, 430 fed females were dissected and checked for *Leishmania* infections eight days post blood meal (PBM) to allow the development of mature infections (Table 1). The average infectiousness of mice to feeding *P. orientalis* females steadily increased from week 2 p.i. (15% of sand flies positive) to week 8 p.i (22% of sand flies positive), however, the differences were not statistically significant. Sand fly infections were characterized by dense parasite colonization of the stomodeal valve in 76% (65/ 86) of the infected *P. orientalis* females indicating these females were competent to transmit the parasites by bite [19].

**Table 1:** **Infectiousness of BALB/c mice for** ***Phlebotomus orientalis*** **feeding on inoculated ears: first series of xenodiagnoses**

**Mouse No.**	#1 and #2*		**#3 and #4**		**#5 and #6**		**#7 and #8**		**#9 and #10**		**Total**	
**Weeks p.i.**	**No fed females**	**No (%) positive**	**No fed females**	**No (%) positive**	**No fed females**	**No (%) positive**	**No fed females**	**No (%) positive**	**No fed females**	**No (%) positive**	**No fed females**	**No (%) positive**
2	7	0 (0)	7	1 (14.3)	11	2 (18.2)	8	2 (25.0)	0	-	33	5 (15.2)
4	0	-	14	1 (7.1)	17	4 (23.5)	20	5 (25.0)	20	3 (15.0)	71	13 (18.3)
6	58	11 (19.0)	69	12 (17.4)	30	6 (20.0)	37	8 (21.6)	30	8 (26.7)	224	45 (20.1)
8	15	3 (20.0)	14	5 (35.7)	39	6 (15.4)	13	3 (23.1)	21	6 (28.6)	102	23 (22.5)
Total	80	14 (17.5)	104	19 (18.3)	97	18 (18.6)	78	18 (23.1)	71	17 (23.9)	430	86 (20.0)

Sand flies were dissected and checked under a light microscope 8 days post feeding on the mouse ear (site of *L. donovani* inoculation).

p.i., post infection; *, two BALB/c mice were exposed to sand flies in one cage.

In four of five mice sacrificed on week 8 p.i., qPCR demonstrated the highest parasite numbers in the inoculated ears and their draining lymph nodes with fewer parasites found in the spleens. In one of these mice, parasites were also detected in the liver. In the fifth mouse, the highest parasites burden was found in the spleen and, in contrast to other mice, parasites were also found in the blood. Other organs tested (contra-lateral ears and their draining lymph nodes, paws and tails) were negative in all five mice (Table 2).

**Table 2:** **Presence of** ***Leishmania donovani*** **DNA in various tissues of BALB/c mice**

	**Mice sacrificed 8 weeks p.i.**					**Mice sacrificed 15 weeks p.i.**				
	**#3**	**#4**	**#6**	**#8**	**#10**	**#1**	**#2**	**#5**	**#7**	**#9**
Inoculated ear (IE)	++	+++	++++	++++	neg.	+	++	+++	++++	++++
Contralateral ear (CE)	neg.	neg.	neg.	neg.	neg.	neg.	neg.	neg.	+	++
Draining lymph node of the IE	+++	+++	++++	++++	neg.	neg.	neg.	++++	+++++	+++++
Draining lymph node of the CE	neg.	neg.	neg.	neg.	neg.	neg.	neg.	neg.	neg.	neg.
Paws	neg.	neg.	neg.	neg.	neg.	neg.	neg.	neg.	neg.	neg.
Tail	neg.	neg.	neg.	neg.	neg.	neg.	neg.	neg.	neg.	neg.
Spleen*	+	++	+	++	+++	neg.	neg.	++	++	++
Liver	neg.	neg.	neg.	+	neg.	neg.	neg.	+	++	++
Blood	n.d.	n.d.	neg.	neg.	++	n.d.	n.d	neg.	neg.	neg.

*Spleens of mice 5, 7 and 9 were positive also by parasite cultivation; in other mice cultivation attempts were not done.

Quantity of parasites was tested by qPCR and scored as + (<10^2^), ++ (10^2^-10^3^), +++ (10^3^-10^4^), ++++ (10^4^-10^5^), +++++ (>10^5^).

neg., negative, n.d., not done; p.i., post infection.

### Xenodiagnosis experiment II: week 9 – 15 post inoculation

Sand flies were allowed to feed on different body parts of five *L. donovani*-infected anaesthetized mice. Engorged females were sorted into groups having fed on (a) inoculated ears, (b) contralateral ears, (c) front paws (d) hind paws with tails. In total, 1,314 sand flies were tested by qPCR on day 2 PMB for the presence of *Leishmania* DNA.

The percentage of sand fly females that became infected by feeding on the inoculated ears of mice, ranged from 0 to 45%. Parasite numbers extrapolated from the qPCR values, of females tested on day 2 PBM, ranged between 10 and 4x10^4^ with median 250 per infected sand fly (Table 3). From week 9 through week 12 p.i. the infectiousness to sand flies that fed on the inoculated ear did not differ significantly; the percentage of females that became infected ranged from 12.5% to 24.4% (p = 0.2). On the contrary, on week 15 p.i. such infection rate decreased significantly (6.3%; p = 0.01). By week 12 p.i. some sand flies feeding on the contralateral ears of two of the mice also became infected, however on week 15 p.i. no *Leishmania* DNA was detected in pools of females that fed on the contralateral ear. Sand flies feeding on paws or tails were not infected throughout the experiment (Table 3).

**Table 3:** **Infectiousness of BALB/c mice for** ***Phlebotomus orientalis*** **: second series of xenodiagnoses**

**Mouse No.**	**Site of feeding of sand flies**	**Weeks post-infection**									
		**9**		**10**		**11**		**12**		**15**	
		**No fed females**	**No (%) positive**	**No fed females**	**No (%) positive**	**No fed females**	**No (%) positive**	**No fed females**	**No (%) positive**	**No fed females**	**No (%) positive**
#1	Inoculated ear	13	1 (7.7)^a^	9	0	21	3 (14.3)^b^	15	1 (6.7)^c^	6	0
	Contralateral ear	3	0	13	0	6	0	18	0	5	0
	Front paws	8	0	7	0	4	0	9	0	9	0
	Hind paws and tail	28	0	7	0	12	0	9	0	10	0
#2	Inoculated ear	20	4 (20.0)^d^	6	0	12	1 (8.3)^e^	14	0	8	0
	Contralateral ear	4	0	16	0	10	0	13	0	5	0
	Front paws	7	0	6	0	9	0	14	0	15	0
	Hind paws and tail	4	0	5	0	3	0	9	0	10	0
#5	Inoculated ear	23	5 (25.0)^f^	28	3 (10.7)^g^	27	10 (37.0)^h^	19	5 (26.3)^i^	41	3 (7.3)^j^
	Contralateral ear	11	0	24	0	40	0	12	0	10	0
	Front paws	3	0	17	0	17	0	13	0	11	0
	Hind paws and tail	19	0	15	0	29	0	5	0	10	0
#7	Inoculated ear	20	2 (13.3)^k^	18	2 (11.1)^l^	16	0 (0)	18	6 (33.3)^m^	11	1 (9.0)^n^
	Contralateral ear	14	0	22	0	20	0	8	pos.	14	0
	Front paws	3	0	7	0	9	0	6	0	5	0
	Hind paws and tail	0	0	26	0	6	0	8	0	10	0
#9	Inoculated ear	15	8 (34.7)^o^	35	7 (20.0)^p^	18	4 (22.2)^q^	20	9 (45.0)^r^	13	1 (7.7)^s^
	Contralateral ear	20	0	12	0	17	0	27	pos.	8	0
	Front paws	9	0	6	0	3	0	9	0	8	0
	Hind paws and tail	8	0	23	0	9	0	32	0	5	0
∑	Inoculated ear	91	20 (22.0)	96	12 (12.5)	94	18 (19.1)	86	21 (24.4)	79	5 (6.3)
	Contralateral ear	52	0	87	0	93	0	78	2/5 pools	42	0
	Front paws	30	0	43	0	42	0	51	0	48	0
	Hind paws and tail	59	0	76	0	59	0	63	0	45	0

Engorged sand flies were maintained on 50% aqueous sucrose solution till day 2 post-blood meal at 26°C and then tested by qPCR for presence and quantity of *Leishmania donovani* DNA. Sand flies fed on the inoculated ear were tested individually; sand flies fed on other parts of mice body were pooled for PCR analysis.

p.i., post infection, pos. = positive pool, a-s, numbers of *Leishmania* detected by qPCR: a, 320; b, all <10; c, <10; d, 1652, 419, 643, 297; e, 430; o, 708, 21, 245, 27, 476, 4035, 24, 68; p, 1850, 211, 39, 3216, 74, 48, 162; q, 18, 143, 2161, 614; r, 20, 481, 5031, 50, 21, 171, 572, 228, 17; s, 279; f, 153, 113, 168, 404, 665; g, 69, 1286, 517; h, 1574, 37, 30, 11085, 1021, 15, 2844, 137, 64, 1815; i, 425, 100, 444, 1917, 2186; j, 273, 860, 50; k; 143, 180; l, 60, 1333; m, 146, 382, 40049, 66, 151, 1123; n, 256.

Although all through the experiment, none of the mice showed any sign of disease, following their sacrifice (week 15 p.i.), PCR showed that all five mice harbored parasites in the inoculated ears (Table 2). In three mice, parasites were also found in the corresponding draining lymph nodes (very high burdens), as well as the spleen and liver. In the two mice in which the contralateral ears became infectious to biting sand flies, parasite DNA was detected in them as well. Mice with higher parasite numbers in the ears (5, 7 and 9) were more infectious to sand flies than mice with lower parasite burdens (1 and 2).

## Discussion

We used BALB/c mice to perform xenodiagnosis of *L. donovani* infections in order to establish tools for studying the putative role of asymptomatic parasite carriers in the transmission dynamics of VL. BALB/c mice are classified as susceptible to *L. donovani* with the capacity to heal visceralizing *Leishmania* infections spontaneously (reviewed by [20]). Thus, in our study BALB/c mice represented asymptomatic humans that comprise the majority of *L. donovani* infections encountered in endemic areas [20] and not the relatively rare, patent VL cases. BALB/c mice infected with *L. donovani* developed persistent burden of parasites at the site of inoculation, draining lymph nodes and internal organs with only limited dissemination to the contralateral ears. Ear tissues and their draining lymph nodes exhibited heavy parasite burdens, with parasites in lymph nodes often outnumbering those found in ears. Since parasites were not normally detected in blood, we assume that the relevant parasite burdens are those in the skin and are almost exclusively localized close to the site of the infective bite. It is not known whether skin burdens of *L. donovani* in asymptomatic humans are also restricted to the region of the infectious bite. One indication that this may be the case derives from PKDL patients in whom parasite-laden nodules are normally more common in the limbs, neck and face – body parts exposed to biting sand flies [21]. Studies on *L. infantum*, the causative parasite of VL in Latin America, demonstrated high parasite densities in the skin (higher than lymph nodes or viscera) in both symptomatic and asymptomatic dogs [22]. Furthermore, a strong correlation between the parasite load in ear tissue and the infectiousness to *Lutzomyia longipalpis*, the main vector species, was demonstrated in symptomatic dogs in Brazil [23,24].

Tissue tropisms and host virulence of *Leishmania* parasites is influenced by many factors. An important role is played by *Leishmania* species-specific genes [25], but the mode of infection (intradermal *vs*. intravenous), the size of the parasite inoculum as well as the *Leishmania* life-cycle stages (procyclic *vs*. metacyclic) used, all influence the outcome of infection. VL initiated by sand fly bite has not been studied in mice but has recently been described in hamsters where VL progressed slowly resembling the chronicity of the disease in humans [26]. However, most of the experiments with BALB/c mice employed the intravenous route of infection, resulting in rapid visceralization, unlike the progression of VL in humans (reviewed by [27]). The intradermal route of infection, used in the current study, is closer to the natural mode of transmission since parasites are exposed to the localized immune responses in the skin [28]. In addition, isolation of parasites from the thoracic midguts of infected sand flies facilitated the inoculation of a known number of predominantly metacyclic stage parasites. This standardized procedure resulted in infections without external signs of disease that visceralized, while the dermal inoculation sites remained infectious to biting sand flies. The percentage of *P. orientalis* infected with *L. donovani* while biting at or close to the site of inoculation (approximately 19%) was similar to that observed for other natural vectors; *L. longipalpis* feeding on symptomatic dogs infected with *L. d. infantum* in Brazil (13 – 28%, [23,29-32]).

Previous studies have demonstrated that progression of *Leishmania* infections in susceptible hosts is influenced by the infective dose [33]. The number of parasites transmitted by sand flies to the host has been shown to be greatly variable [33-35]. The infective dose per mouse used in our experiment (9x10^4^ - 10^5^), was significantly lower than inoculums normally delivered by needle-injection (10^6^-10^9^ parasites, reviewed by [36]) and equaled or only slightly exceeded the upper range detected in experiments with *P. duboscqi* transmitting *L. major* (10^5^ parasites inoculated per bite [33]) and *P. perniciosus* and *L. longipalpis* transmitting *L. infantum* (4x10^4^ and 10^4^ parasites per bite, respectively) [34,35]. Importantly, this infective dose comprised 65–73% metacyclics which closely approximate the mean percentage of *L. donovani* and *L. infantum* metacyclics transmitted by *L. longipalpis* (66-82%) [26].

Transmission of *L. donovani* is assumed to be chiefly anthroponotic unlike *L. infantum* that circulates as a classical zoonosis with dogs serving as the main reservoir hosts [37]. However, recent findings suggest that reservoir animals may contribute to the transmission of *L. donovani* as well. For example, high levels of seroprevalence and confirmed infections with *L. donovani* were found in dogs in Sudan [38] while qPCR results indicated the possibility that goats were infected with *L. donovani* in Nepal and India [39]. Finding PCR positive animals does not necessarily mean they serve as parasite reservoirs for biting sand flies. Indeed, such animals may simply serve as parasite sinks, i.e. animals upon which infected sand flies feed but do not contribute to their infection. Therefore, animal models for studying *L. donovani* development in the host and its subsequent infectiousness to sand flies, warrant in-depth studies in the laboratory and in the field.

BALB/c mice are well established animal models for studying host-parasite interactions, pathogenesis, immunological responses and vaccines against VL [20,36]. Our study established that BALB/c mice infected with *L. donovani* did not develop external signs of disease and are highly infectious to biting sand flies, making them a suitable laboratory animal for xenodiagnostic studies as well. The fact that apparently healthy ear tissue maintains sufficient quantities of *L. donovani* amastigotes to induce heavy infections in sand fly vectors, points to the need for further studies evaluating wild rodents in endemic foci as potential reservoir hosts for *L. donovani*.

## Conclusions

This is the first paper to characterize the infectiousness profile for biting sand flies of hosts without external signs of disease infected with *L. donovani*. We established here an animal model using BALB/c mice inoculated intradermally with infective-stage (metacyclic) parasites isolated from experimentally infected sand flies. This approach enabled the counting of exact numbers of parasites in the inoculums while the site of inoculation and parasite forms used, remained similar to those delivered during infected sand fly bites. Experimentally infected mice harbored *L. donovani* parasites for many weeks without showing any apparent symptoms. They developed persistent parasitaemias at the site of inoculation, draining lymph nodes and internal organs with only limited dissemination to the contralateral ears. Although, like asymptomatic human cases, infected mice did not show signs of disease, they were highly infectious to *P. orientalis*, the vector of VL in north Ethiopia and Sudan. The study showed that BALB/c mice comprise a suitable laboratory model for performing xenodiagnosis of *L. donovani* infections and warrants the consideration of rodents as potential reservoir hosts of *L. donovani*. Data about temporal and spatial patterns of infectiousness of *L. donovani* infected mice for sand flies showed that parasites persist in the inoculation site and are found transmissible for months to sand flies biting on the same site. This finding may promote improved understanding of the transmission dynamics and epidemiology of VL.

## Abbreviations

VL: Visceral leishmaniasis
CL: Cutaneous leishmaniasis
LST: Leishmanin skin test
PKDL: Post-kala-azar dermal leishmaniasis
PBM: Post bloodmeal
p.i.: Post infection
neg.: Negative
n.d: Not done
